# The mental-health patient-activation measure: assessing validity, reliability, and responsiveness in outpatient settings

**DOI:** 10.1186/s12888-025-06939-5

**Published:** 2025-05-22

**Authors:** Tatiana Skliarova, Mariela L. Lara-Cabrera, Mathias Forsberg Brobakken, Jørn Heggelund, Einar Vedul-Kjelsås, Ismail Cüneyt Güzey, Hege Hafstad, Solveig Klaebo Reitan, Mona Nygård

**Affiliations:** 1 Department of Mental Health, Faculty of Medicine and Health Sciences, Norwegian University of Science and Technology (NTNU), Trondheim, NO-7491 Norway; 2 Department of Mental Healthcare, St. Olavs Hospital, Nidelv Community Mental Health Center, Trondheim University Hospital, Trondheim, Norway; 3 Department of Psychosis and Rehabilitation, Department of Mental Healthcare, St. Olavs Hospital, Trondheim University Hospital, Trondheim, Norway; 4 Faculty of Health Sciences and Social Care, Molde University College, Molde, Norway; 5 Regional Center for Healthcare Improvement, St. Olavs Hospital, Trondheim University Hospital, Trondheim, Norway; 6 Department of Research, Innovation and Education, Department of Mental Healthcare, St. Olavs Hospital, Trondheim University Hospital, Trondheim, Norway; 7 Vårres Regional User-Led Center Mid-Norway, Trondheim, Norway

**Keywords:** Mental health; psychometric properties, Exercise training implementation, Mental disorders, Patient activation, Patient-centred care, Patient participation, User involvement, Validation studies, Participation in research

## Abstract

**Introduction:**

The patient activation measure–mental health (PAM-MH) scale, adapted from the Patient Activation Measure-13 Items (PAM-13) developed in the United States, was designed to assess patient engagement and activation in mental health care. Despite initial validation of the PAM-MH in its original context, its applicability in other settings and its comparison to the gold standard PAM-13 remain unexplored. Furthermore, research addressing the face and construct validity of the PAM-MH is limited. This multi-phase study evaluated the face validity, construct validity, reliability and responsiveness of the PAM-MH in a Norwegian outpatient setting.

**Methods:**

A participatory approach was adopted to actively involve user representatives in validating the scale. The validation process consisted of four interconnected studies. In the first sub-study, user representatives were invited to contribute to establish face validity by providing quantitative feedback on the perceived value and burden of completing the PAM-MH. The second sub-study examined the construct validity. It was hypothesised that the PAM-MH measures the construct of patient activation and would therefore correlate with the PAM-13. This hypothesis was tested using Pearson’s *r* in a sample of 55 outpatients. The third sub-study evaluated test–retest reliability (via ICC) and internal consistency (using Cronbach’s α), in a sample of 27 outpatients who completed the PAM-MH on two separate occasions. The final sub-study explored the responsiveness of the scale to change, guided by predefined hypotheses, in a sample of 11 outpatients.

**Results:**

Adequate acceptability was established from users’ views of the value and burden of the scale (overall mean “value” score was 59.7% and mean “burden” was 39.6%). All hypotheses, established a priori for construct validity, reliability, and responsiveness-to-change, were confirmed.

**Conclusion:**

This multi-phase study employed a participatory approach to validate the PAM-MH in a Norwegian outpatient context. Preliminary results demonstrated satisfactory face validity and construct validity, along with good reliability and responsiveness-to-change. The findings suggest that the PAM-MH is both valid and reliable, making it a suitable questionnaire to measure patient activation in a Norwegian outpatient setting.

**Supplementary Information:**

The online version contains supplementary material available at 10.1186/s12888-025-06939-5.

## Introduction

“Patient activation” refers to the knowledge, skills, and confidence that enable patients to actively participate in their medical and other health care [1]. It is central to both the consumption of health services and the concept of treatment of chronic diseases [2]. Because people with chronic diseases need continued access to care and treatment, they account for a significant proportion of healthcare costs. Therefore, if functioning can be maintained through higher levels of patient activation, this can benefit both patients and wider society [3]. Patients with high activation tend to have fewer mental symptoms, better adherence to treatment, and faster recovery [4, 5]. Studies have shown that patient activation is a predictor of important health-related practices, including self-management and health-information seeking [3]. Studies have also indicated that individuals diagnosed with schizophrenia reporting higher levels of activation are likely to have better insights into their illness and exhibit improved recovery and adherence to medication regimens [5, 6]. In addition, amongst individuals with serious mental illness, patient activation is inversely correlated with healthcare costs [7]. Patients with high activation are also more likely to participate in self-care activities [8] and to be involved in their treatment [6, 9]. However, patients with mental illness may face additional barriers to engagement in their care, such as cognitive impairment and negative symptoms [10], as well as struggling to engage in healthy behaviour. Thus, measuring patient activation provides a way to assess level of engagement in the healthcare process. Doing so for individuals with schizophrenia could provide invaluable insights for healthcare providers and researchers into these patients’ level of engagement in their treatment and healthcare.

The patient activation measure scale, consisting of 13 items (PAM-13), was initially developed to measure activation in patients with chronic physical conditions. Activation was considered a broad concept [1]. Measured by the PAM-13, it can be divided into four levels of development: (1) patients feel passive about their health and need more time to be ready to play an active role in maintaining and ensuring their own health. (2) Patients feel uncertain and lack knowledge about their health. (3) Patients can take actions aimed at maintaining their health, but they need more skills and confidence to support such behaviour. (4) Patients try to adhere to behaviours which support their health, but when faced with stress and life difficulties, they are not ready to support such behaviours [11]. Most validation studies supported the construct validity of the PAM-13, but more research is needed, in particular to explore the content validity [12].

A patient’s health problems can be complex and multifactorial, and a patient’s activation in their own health management may differ for physical health issues compared to mental health issues. The original PAM-13 was developed to investigate patient activation regarding physical health. Green et al. [4] identified the need to investigate this specifically for mental health and thus modified the scale, proposing the Patient Activation Measure–Mental Health (PAM-MH). The PAM-MH is identical to the PAM-13, with the word ‘health’ replaced by ‘mental health’. The psychometric properties of the PAM-MH were assessed using several methods, including Rasch analysis, test–retest reliability, and concurrent and construct validity. Construct validity was investigated by examining the relationships between the PAM-MH scores and other measures of mental health, patient engagement (recovery assessment), and quality of life. The reported test–retest reliability was good [4]. Concerning validity, the PAM-MH demonstrated a positive relationship between recovery and the physical and mental domains of quality of life [4]. Although the PAM-MH is a promising scale, validation studies are still required in countries outside of the United States. Furthermore, the construct validity of the PAM-MH has yet to be systematically compared with that of the original PAM-13 scale. As there are plans to employ the PAM-MH in clinical trials [13, 14], there is a need to ensure its psychometric properties in other societies.

User involvement in research is intended to enhance the relevance and utility of studies for end-users. Through participatory research approaches [15], studies are designed to improve the quality and applicability of findings by incorporating the unique lived experiences and perspectives of service users. In mental-health research, user involvement plays a critical role in improving services; empowering service users; and integrating their knowledge, perspectives, and expertise throughout all stages of the research process [15, 16]. User-researchers or co-researchers can contribute at various levels of the research process [16], employing diverse methodological approaches, such as qualitative studies [17, 18], experimental design [19–21], and scale validation [22–26]. For instance, when developing a scale to assess experiences of activation and engagement in mental-health services, the involvement of user representatives, co-researchers and experts with lived experience is crucial. Their contributions are instrumental for evaluating the scale’s content, identifying its practical value, and assessing the burden associated with self-rated questionnaires [23]. Such partnerships between researchers and lived-experience experts emphasise the importance of creating interventions that are relevant and meaningful to their intended users. However, despite the availability of scales to measure patient activation and engagement, little research has focused on participatory approaches to scale development and validation. Although the PAM-MH was initially validated in an American mental-health context, its development did not involve user representatives. Additionally, its applicability in other settings and its comparison with the gold standard PAM-13 have yet to be thoroughly investigated. Research on the face and construct validity of the PAM-MH has been limited. To address these gaps, this multi-phase study adopted a participatory approach to validate the PAM-MH in a Norwegian outpatient context.

### Objectives

The first objective was to validate whether the PAM-MH accurately reflects the perspectives of those with lived experience (face validity). This was tested by involving 16 experts and user representatives, each of whom reviewed PAM-MH and provided feedback through a questionnaire on the measure’s value and burden. The second objective was to verify that the PAM-MH effectively measures what it is intended to measure (construct validity). This was done by comparing it to the established PAM-13 using Pearson’s r, using data collected from 55 outpatients. The third objective was to assess the consistency of the PAM-MH over time and measure its internal consistency (reliability). This involved evaluating test–retest reliability (ICC) and Cronbach’s α. The final objective was to determine the measure’s ability to detect changes over time (responsiveness). This was investigated using predefined hypotheses in a sample of 11 outpatients.

## Methods

### Participatory approach

A participatory approach was employed to engage user representatives in the validation of the PAM-MH into Norwegian. We followed a three-stage translation process when developing the Norwegian version of the PAM-MH questionnaire. This process was adapted from Sousa & Rojjanasrirat, 2010 [27]. The first step involved forward translation, in which two professional bilingual translators independently translated the original PAM-MH English version into Norwegian. During this stage, one discrepancy was reported, as the term “mental health” was translated in two different ways: “psychiatric” and “mental.” After discussion with the research team, a consensus translation was reached, opting for the term “mental” to enhance simplicity and clarity. The second step involved back-translation and review, in which one independent translator, unaware of the original PAM-MH version, back-translated to English. The back-translated version was then reviewed by a multidisciplinary research team comprising clinicians and a user representative. Through iterative discussions, the PAM-MH was evaluated for clarity and conceptual equivalence. The third and final step was a pilot test conducted among ten user representatives recruited via a user-led mental health organisation. The representatives were selected from a user-led organisation in Mid Norway and anonymously evaluated the translation using a paper version of the scale. Participants discussed and assessed the comprehensibility and appropriateness of the translated items. No significant issues were reported, resulting in the establishment of the finalized PAM-MH Norwegian version. In the next phase, two user representatives were involved in the study design: one led the data collection for face validity, and the other participated in collecting data on responsiveness-to-change by engaging with outpatients over a 12-week period. At the end of this process, the co-researcher was involved in the dissemination phase. The guidelines for reporting involvement of patients and the public involvement short-form (GRIPP2, Appendix S1) were used [28, 29].

### Study design and data collection

**Fig. 1:**
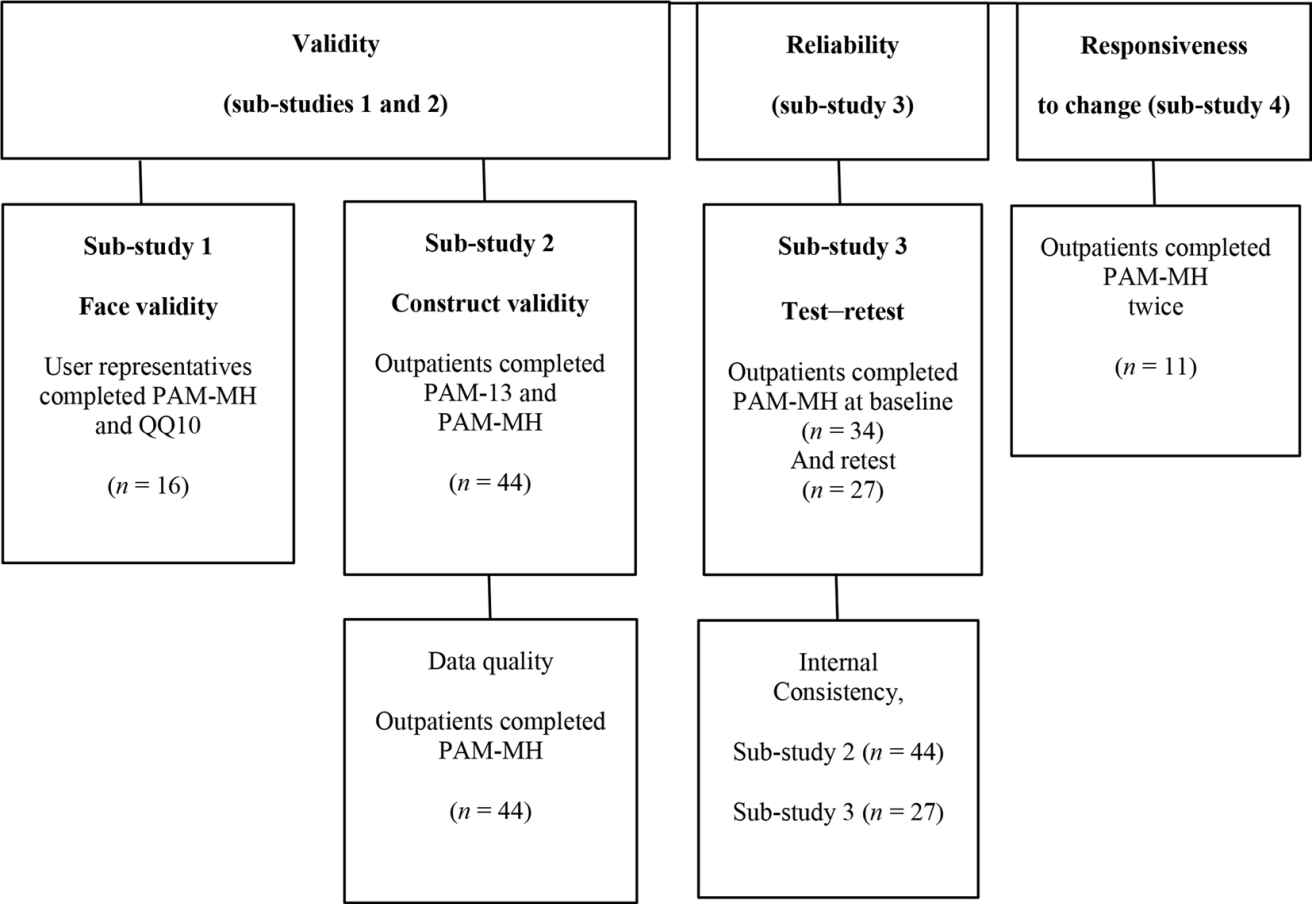
Flow-chat of the study

The validation process consisted of four interconnected studies (Fig. 1).

#### Sub-study 1. Face validity

In the first sub-study, we examined the face validity of PAM-MH. The data collection was planned and conducted in collaboration with user representatives from the Norwegian organisation Vårres Regional User-led Center Mid-Norway, and five mental-healthcare professionals. Participation was anonymous. All user representatives were provided with both oral and written information about the study. Informed consent was obtained from all participants through their anonymous completion of the questionnaires and the return of the sealed envelope at the conclusion of the group meeting. No personal information, names, or other identifying details were collected, and strict measures were taken to ensure confidentiality and anonymity throughout.

User representatives from user-led organisations were recruited by a co-researcher (HH). A total of 17 adults were invited to participate in a group meeting. Sixteen user representatives took part in this sub-study: nine men, five women, and two individuals who did not report their gender. The majority of participants were aged between 40 and 54 years old.

Participants completed the PAM-MH, alongside the QQ-10, a 10-item questionnaire designed to assess the perceived value and burden of using the PAM-MH. The involvement of user representatives was critical for ensuring that the PAM-MH accurately captured the perspectives of those with lived experience. Additionally, the QQ-10 provided feedback on the measure’s feasibility. After completing both scales, participants returned their responses in sealed envelopes to ensue confidentiality.

#### Sub-study 2. Construct validity

In the second sub-study, outpatients diagnosed with schizophrenia spectrum disorders (ICD-10 (International Classification of Disease), F20-29) were invited to complete the PAM-MH and PAM-13 scales. Outpatients were recruited by a project coordinator from the municipality’s supportive housing facilities or through therapists at the local community health clinic. Patients who were interested were invited to visit the clinic for more information before deciding to participate. The data collection was completed as part of a more extensive multimethod study [30, 31] on measuring and enhancing patient activation. The study was approved by the Regional Committee for Medical and Health Research Ethics in Norway (REC number 2015/1611), and all patients signed an informed consent form before any data were collected. A user representative was actively involved in this project, taking part in the planning of the project, execution, and data collection.

A total of 55 patients were recruited (31 men and 24 women), and the mean age was 35 ± 11 years (age range of 20–59 years). Thirty-three patients had paranoid schizophrenia, and the others were on the schizophrenia spectrum. At baseline (T0), 41 patients completed PAM-13 and 44 completed PAM-MH.

#### Sub-study 3. Reliability

The third sub-study assessed test–retest reliability and Cronbach’s α. Participants for the test–retest sample were invited to complete the PAM-MH through healthcare professionals working at the clinic. The recruitment was via a project coordinator from the municipality’s supportive housing facilities, and through therapists at the local community-health clinic. All outpatients received both oral and written information about the study, and informed consent was obtained from all participants.

After providing consent, outpatients were asked to complete the PAM-MH at a training clinic in a mental-health hospital. A total of 34 patients (18 men and 16 women, aged 35.0 ± 11.9 years, an age range of 20–58 years) consented to participate, with 27 outpatients (17 men and 10 women, aged 34.6 ± 11.9 years, an age range of 20–58 years) completing the PAM-MH on two separate occasions. In the group of 34 patients, 17 had paranoid schizophrenia and the others were on the schizophrenia spectrum. For the group of 27, 14 had paranoid schizophrenia and the others were on the schizophrenia spectrum. Participants in both groups independently filled out paper-and-pencil versions of the scale while seated in the clinic’s waiting area. Outpatients in the Cronbach’s α sample were those participating in sub-study 2 and sub-study 3.

#### Sub-study 4. Responsiveness-to-change

This sub-study assessed the responsiveness of the PAM-MH scale to changes in patient activation following an intervention to encourage independent exercise in individuals with schizophrenia spectrum disorders. An exercise intervention was chosen due to clinical experience suggesting various benefits of exercise, namely improved cognitive function [32] and reduced mental-health symptoms [33]. The user representatives involved in this study wished to investigate and promote exercise as a non-pharmacological intervention that could trigger changes in patient activation.

The recruitment and assessments in sub-study 4 followed the same in-person procedures used in sub-study 3. Outpatients who were diagnosed with schizophrenia spectrum disorders and who met the inclusion criteria and had volunteered for exercise training were included in the sub-study. Prior to inclusion, participants were provided with both oral and written information about the study. Participants in this sample received treatment as usual, along with two supervised exercise training sessions that included support and education on how to exercise. They were invited to complete the PAM-MH questionnaire both before the training (T0) and after 12 weeks (T2). Of the 18 outpatients who expressed interest in participating, 11 completed the PAM-MH at T2. The training and assessments were conducted at the Exercise Training Clinic, an outpatient psychiatry clinic at St. Olavs Hospital.

### Outcome measures

The questionnaire QQ-10 was used in sub-study 1. The QQ-10 consists of 10 items, designed to provide quantitative data on the perceived value and burden of answering self-reported scales and questionnaires. Each item is scored on a Likert scale, where “0” means “strongly disagree” with the statement and “4” means “strongly agree” [34]. The scale consists of two domains. The first domain includes six questions related to the positive values of the scale (helps to communicate about the condition, is relevant to the condition, and includes all the aspects of the condition that a patient may be concerned about, as well as whether the scale is easy and enjoyable to complete, and the participants would be happy to complete it again as part of treatment). The second domain asks four questions about the negative burden of completing the scale (too long, embarrassing, complicated, upset me). The QQ-10 serves as a standardised brief measure of face validity [34], aimed to be used as a quick intuitive first step to identify whether the PAM-MH seems appropriate before more rigorous forms of validity are conducted. In this research, Cronbach’s α = 0.609 for the whole scale.

The PAM-13 scale, developed in 2005, is a generic self-report scale for measuring patient activation in people from the general population aged 45 years and older [3, 10]. It consists of 13 items, scored from 1 (“strongly disagree”) to 4 (“strongly agree”) or 0 (“not applicable”). The scale includes questions related to confidence in taking actions to prevent or minimise symptoms and problems associated with the health condition, knowledge of what each prescribed medication does, and the confidence to determine when to seek medical care and when to handle a health problem independently. The PAM-13 serves as a standardised measure of a patient’s readiness to manage their health, which can help healthcare providers tailor interventions accordingly. The scoring algorithm of the scale is provided by the company Insignia Health [35], which licences the PAM questionnaire. The scale ranges from 0 to 100, with a lower score indicating a lower level of patient activation and a higher score a higher likelihood of patient engagement in the treatment process.

The PAM-MH is an adapted version of the PAM-13 scale for mental-health population, using data from a large sample of patients from a not-for-profit integrated health-plan service in the United States [4]. It was developed in 2009 to assess self-management in adults diagnosed with severe mental-disorders and consists of 13 questions (see Table 1). The items were designed to assess an individual’s knowledge, skills, and confidence in relation to managing their mental health. Each item is scored on a Likert scale, where 1 means “strongly disagree” and 4 “strongly agree”. The scale consists of a single domain that evaluates the extent to which individuals feel informed, engaged, and capable of managing their mental health. The PAM-MH includes questions on the confidence needed to take action to prevent or minimise symptoms or problems associated with a mental-health condition, knowledge about how to prevent further mental-health problems and about different treatment options available for mental-health conditions, as well as the confidence to determine when to seek professional help and when to handle a mental-health issue independently.

**Table 1:** Item descriptive for the PAM-MH (test–retest sample, *n* = 27)

	Mean T0 (SD)	Mean T1 (SD)
1.When all is said and done, I am the person who is responsible for managing my health condition	3.04 (0.56)	3.11 (0.75)
2. Taking an active role in my own health care is the most important factor in determining my health and ability to function.	3.19 (0.66)	3.37 (0.49)
3. I am confident that I can take actions that will help prevent or minimise some symptoms or problems associated with my health condition	3.07 (0.73)	3.15 (0.60)
4. I know what each of my prescribed medications does	2.93 (0.55)	2.93 (0.61)
5. I am confident I can tell when I need to go get medical care and when I can handle a health problem myself.	2.89 (0.69)	2.93 (0.67)
6. I am confident I can tell my health provider the concerns I have even when he or she does not ask.	3.07 (0.73)	3.07 (0.73)
7. I am confident I can follow through on the medical treatment I need to do at home.	2.74 (0.59)	2.81 (0.74)
8. I understand the nature and causes of my health condition.	3.00 (0.73)	2.89 (0.64)
9. I know the different medical treatment options available for my health condition.	2.63 (0.74)	2.96 (0.65)
10. I have been able to maintain the lifestyle changes I have made for my health.	2.81 (0.62)	2.78 (0.51)
11. I know how to prevent further problems with my health condition.	2.74 (0.66)	2.81 (0.68)
12. I am confident I can find a solution when new situations or problems arise with my health condition.	2.81 (0.56)	2.74 (0.81)
13. I am confident I can maintain lifestyles changes, like diet and exercise, even during times of stress	2.63 (0.88)	2.70 (0.72)
Patient activation from 0 to 100, total mean.	53.56 (11.17)	55.16 (12.61)

Note. Individual items response options ranged from 1–4. SD = Standard deviation; T0 = test; T1 = retest

The PAM-MH serves as a standardised measure of a patient’s readiness to manage their mental health, which can help clinicians tailor interventions accordingly. We scored the PAM-MH in the same way as PAM-13 (transforming the total score from 0 to 100). A total score was calculated only if at least 10 of 13 items had been answered.

### Statistical analyses and hypotheses

SPSS (v. 22, CA, USA) was used for data analysis and Microsoft Excel (365 Desktop) to create the figure. Descriptive statistics (percentages, means and standard deviations) were used for face validity analysis, item analysis and sociodemographic variables.

#### Sub-study 1. Face validity

To assess face validity using the QQ-10, we calculated the overall mean values for the value domain and the burden domain separately. The QQ-10 scores range from 0 to 100, with 0 being the worst and 100 being the best. We hypothesised that the QQ-10 would yield high scores for value (greater than 50%) and low scores for burden (less than 40%). Additionally, we calculated the frequencies and percentages for responses to each item.

#### Sub-study 2. Construct validity

To evaluate the construct validity, we used Pearson’s *r*. Based on the assumption that the PAM-MH would assess the construct of patient activation, we hypothesised a correlation with the PAM-13. This is in line with a theoretically derived hypothesis concerning the concept of patient activation. Since the PAM-MH is an adaptation of the original PAM-13 and designed specifically to assess patient activation in individuals with mental-health conditions, we a priori hypothesised that the Pearson’s correlation coefficient would be positive and greater than 0.3.

#### Data quality

Data quality was assessed at mean and item levels by calculating floor or ceiling effects for outpatients’ scores. If more than 15% of the patients scored at the extreme upper or lower ends of the means, a floor or ceiling effect was deemed to be present [36].

#### Sub-study 3. Reliability

For test–retest reliability (i.e., intraclass correlation coefficient [ICC], two-way random effects, absolute agreement) to detect an ICC of > 0.50, 22 patients were required [37]. According to established guidelines [38], ICC values below 0.50 are considered indicative of poor reliability, values between 0.50 and 0.75 suggest moderate reliability, values from 0.75 to 0.90 indicate good reliability, and values exceeding 0.90 reflect excellent reliability. Based on these criteria, a threshold of 0.50 was selected for the present study. The internal consistency of the scale was evaluated with the help of Cronbach’s *α* at two-time points. According to the sample size estimation provided by Bonett [39], for internal consistency Cronbach’s *α* = 0.7 with a precision of ± 0.2, and a 95% CI and a scale with 13 items, a sample size of 26 was needed [40].

#### Sub-study 4. Responsiveness-to-change

Responsiveness-to-change was explored using pre–post data by testing a predefined hypothesis [36] and setting a benchmark of 50% of outpatients achieving an improvement. This benchmark of 50% was informed by clinical experience and prior work [36, 41, 42]. Furthermore, according to Insignia Health, even a single-point change in PAM-score is meaningful [35]. Prior work [43] has also shown that a 4-point increase in PAM-score can be considered meaningful, as such a change correlates with the adoption of various self-management behaviours. Therefore, we a priori hypothesised that a 4-point increase is meaningful and that 50% of the patients in the responsiveness-to-change sample would see an improvement of this size.

## Results

### Sub-study 1. Face validity

**Fig. 2:**
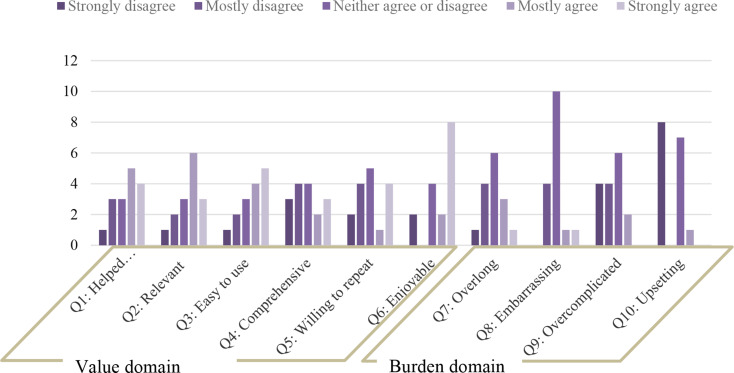
Items’ distribution for each question in the QQ-10

Adequate face validity was established (see Table 2). The QQ-10 results revealed that the overall mean value was 59.7 (*SD* = 28.6), while the mean of the burden score was 39.6 (*SD* = 13.1). The responses of the user representatives are presented in Fig. 2. Of 16 participants, one user representative described the PAM-MH as “partially upsetting,” while half of the user representatives found it enjoyable to complete, and two participants strongly disagreed with this statement. Some user representatives commented that the scale had “many nuances, but still did not cover the topic” or “some similar questions that can be confusing.” Moreover, they recommended adding “an additional scale” that covers predisposing factors, such as “personal and family loads, which can affect coping behaviour.”

**Table 2:** Summary of the construct validity hypotheses

Hypotheses	Results	Decision
Face validity:	“Value” domain > 50% “Burden” domain < 40%	Accept
Construct validity: Correlation between PAM-MH and PAM-13: Pearson’s *r* ≥ 0.3	*r* = 0.77, *p* < 0.001	Accept
Test–retest reliability: ICC is > 0.5 for PAM-MH	*r* = 0.83	Accept
Internal consistency: Cronbach’s *α* > 0.7 for PAM-MH	0.871 in sub-study 2, 0.805 in sub-study 3	Accept
Regarding total mean scores at baseline, no floor/ceiling effects (less than 15% of patients have extreme scores)	No floor or ceiling effect, but 2.3% of patients had the highest possible raw score in PAM-MH, and 4.9% did so in PAM-13	Accept
Responsiveness: More than 50% of patients improve ≥ 4 points following 12 weeks of standard treatment	54.6% improved	Accept

### Sub-study 2. Construct validity

All the hypotheses constructed a priori were confirmed (Table 2). The correlation of the Norwegian version of the PAM-MH scale with the “gold standard” PAM-13 (Norwegian version) was high, *r* = 0.77 (*p* < 0.001).

### Sub-study 3. Reliability

Test–retest reliability, as assessed using the ICC, was also good at 0.83. The internal consistency of the scale was evaluated with the help of Cronbach’s *α* at two-time points (T0 and T1). In both cases, the value was more than 0.70, namely 0.871 and 0.805, respectively. The results are summarised in Table 2.

### Data quality

**Fig. 3:**
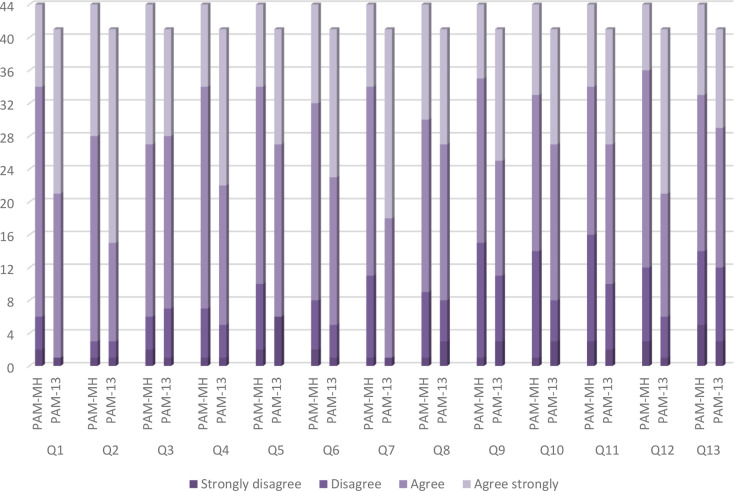
Distribution of PAM-MH and PAM-13 scores at baseline

Floor or ceiling effects would be deemed present if more than 15% of patients gave the lowest or highest possible scores (following the cutoff indicator proposed by Terwee et al. [36]). No floor or ceiling effects were observed for the mean scores. However, all 13 items met the standards for a small floor effect. Furthermore, all individual items exceeded the 15% threshold for ceiling effects– ranging from 18% (8 out of 44 participants answered the highest score in the Item 12) to 38% (17 out of 44 participants answered the highest score in the Item 3). All individual items in the PAM-13 also exceeded 15% for ceiling effects (from 31% in Item 3 to 63% in Item 2). To see the extent of the ceiling and floor effects for the individual items, see Fig. 3.

### Sub-study 4. Responsiveness-to-change

Regarding responsiveness-to-change, we a priori assumed that more than 50% of patients receiving the intervention would improve (≥ 4 points following 12 weeks of standard treatment). In our study, 54.6% of respondents improved their overall PAM-MH score by 4 or more points.

## Discussion

This study evaluated the face validity, construct validity, reliability, and responsiveness of the PAM-MH in a Norwegian outpatient setting, using a participatory approach. The validation process consisted of four interrelated studies. In sub-study 1, user representatives provided feedback on the value and burden of completing the PAM-MH, establishing adequate face validity. In sub-study 2, the construct validity was confirmed through a high correlation (*r* = 0.77) between the Norwegian versions of the PAM-MH and the PAM-13. Sub-study 3 demonstrated good reliability. No floor effects were observed for the mean values, though ceiling effects were noted in some items. In sub-study 4, responsiveness to change was confirmed, as 54.6% of participants improved their PAM-MH score by at least 4 points after 12 weeks of treatment.

QQ-10 was used in our study to determine face validity. The results are comparable to those of previous studies [23, 44–47]. For example, in a study conducted by Twohig et al. (2017), participants rated the questionnaire highly on the “value” domain (mean = 79) and low on the “burden” domain (mean = 21). Moreover, participants commented on its clarity, relevance, and usefulness in reflecting their experiences. Similarly, Gray et al. (2019) found high scores on the “value” domain (mean = 76) and low scores on the “burden” domain (mean = 25) in their development of a questionnaire for hormonal disorders. The authors emphasised the importance of a clear layout and clinical relevance in reducing the burden. However, in the current study, the PAM-MH assessment received a slightly higher score in the burden domain and a slightly lower score in the value domain [44–46]. This outcome may be explained by participants’ concerns about redundancy, the complexity of certain items, and limited coverage, as emphasised in the qualitative feedback. While the instrument is considered acceptable, these observations suggest that the scale’s structure and length may affect participants’ perception of the completion process. These insights, combined with the limited number of user representatives in the current study, may explain the QQ-10 scores in our findings. Furthermore, while the QQ-10 provides standardised quantitative data on the perceived value and burden of self-report scales and questionnaires, it might not capture other psychometric properties, such as content and construct validity. Additionally, the use of advanced qualitative methods, such as semi-structured interviews, could reveal insights through thematic or content analyses. This might restrict our understanding of how outpatients perceive the PAM-MH in the context of their mental health experiences and highlights the need for future qualitative studies to gather more detailed insights into the experiences and perceptions of outpatients. A major strength of this validation study is its participatory approach, which enabled cooperation with user representatives, a co-researcher, and discussions with members of user organisations. This approach was integrated into the design, methodology, and data-collection processes. For instance, user representatives played an important role in the initial selection of the validation scales. After reviewing various self-efficacy and patient-activation measures, user representatives provided critical insights into the relevance of the PAM-MH. The decision to use the PAM-MH as a self-reported outcome measure was influenced by this collaborative process. This participatory decision-making also ensured that the PAM-MH scale would be included in future clinical studies, thereby influencing subsequent research. Consistent with other participatory validation studies [48, 49], our results demonstrate the feasibility of incorporating lived-experience experts into study design and execution. User representatives raised concerns about the burden of completing questionnaires, noting that they could be time-consuming and difficult to complete. By including a user-researcher and representatives from mental-health organisations, the research team was able to streamline the data-collection process, making it more comprehensive and less time-demanding. Thus, we decided to include the QQ-10 in the initial stage of the study. This decision allowed us to balance scientific rigour with the need to minimise the burden of data collection on participants. Moreover, this approach allowed for the adaptation of administration procedures, adding features that reduced participant burden and enhanced validity.

Additionally, the participatory approach fosters co-learning and empowerment processes, as seen in other inclusive research settings [16]. For instance, the researchers gained greater awareness of the context and implications of their activities. This awareness provided the rationale for exploring responsiveness-to-change in patient activation among adults with schizophrenia spectrum disorders through an exercise intervention. A user representative had experienced exercise as having several benefits, such as improved self-efficacy, engagement in self-care, and social engagement. This observation aligned with the construct measures of the PAM-MH, making it an appropriate choice for exploring changes in patient activation. Examination of the links between exercise and activation levels could provide insights into how this intervention could facilitate greater engagement in self-care and treatment. However, it is also important to point out that exercise interventions, while increasingly used, are not common in mental-health settings. Therefore, the detected responsiveness-to-change, while important and in line with previous studies, must be interpreted cautiously.

One of the strengths of our study is the high correlation between PAM-MH and PAM-13, which suggests that PAM-MH retains the foundational elements of patient activation. In line with the hypotheses investigated in the original validation of the PAM-MH, we expected to see strong correlations with congruent concepts because they measure very similar constructs. The strong correlation between PAM-MH and PAM-13 can therefore be considered indicative of conceptual overlap between these two scales. On one hand, this may be due to both questionnaires being based on the same theoretical framework of activation among patients, which includes an individual’s skills, confidence, and knowledge, which help maintain their health. On the other hand, this strong correlation raises questions about whether PAM-MH is capturing distinct content specific to mental health contexts. The intention behind developing PAM-MH was to create a measure that is more specific and nuanced for mental health, and one might expect it to diverge somewhat from the generic PAM-13. This finding suggests that while PAM-MH is effective in measuring patient activation, it may not be sufficiently distinct in the context of mental health. Some research highlights that in both individuals with mental health disorders [5] and chronic disorders [50], patient activation is closely correlated with self-efficacy and faster recovery processes. However, it remains relevant to examine the unique contribution of PAM-MH to specific mental health domains. To address this in future studies, a more comprehensive construct-validation approach should be considered. This approach would include adding scales related to constructs such as self-efficacy, empowerment, recovery scores, and symptom severity to test for concurrent or divergent validity. Incorporating these additional measures would provide a more holistic understanding of PAM-MH’s effectiveness in capturing mental health-specific activation.

The test–retest reliability observed in this study was satisfactory, aligning with previous findings demonstrating good reliability at 14 weeks [4]. This finding is consistent with research evaluating the reliability of the PAM-13 in mental-health settings [48, 49]. Additionally, the internal consistency of the PAM-MH was robust. This finding is comparable to the those of Green et al. [4] and other studies assessing the PAM-13 for patients with substance-use disorders [48]. Our findings, combined with those of the original validation study [4], indicate that the PAM-MH is a reliable questionnaire for assessing patient activation in mental health contexts. However, while the PAM-MH demonstrated strong internal consistency, with a Cronbach’s alpha ranging from 0.81 to 0.97, the higher end of this range may indicate potential redundancy among items. A Cronbach’s alpha exceeding 0.9 can suggest an excess of highly correlated items, rather than high internal consistency. It also highlights the importance of balancing internal consistency with the scale’s capacity to capture distinct facets of the construct it aims to measure.

Consistent with the work of Green et al. [4], the Norwegian version of the PAM-MH is effective in capturing change. More than half of the patients who completed the PAM-MH at 12 weeks had improved their score by more than four points, in accordance with Turner et al. [43] and the hypothesis. The choice of a 50% benchmark for patients achieving this change might be optimistic. These thresholds might need adjustment to account for natural variability in response rates, especially in a population with schizophrenia spectrum disorders and outpatients with severe mental disorders, potentially overestimating the intervention’s impact. Future studies should further investigate the promising improvement in patient activation observed in this intervention. Moreover, any improvement in the PAM-MH score over time could be compared with improvements measured by other tools to verify the responsiveness-to-change over long periods.

Although there is increasing research interest in measuring and validating patient activation in physical conditions, less attention has been given to addressing activation among outpatients with severe mental disorders. As a result, the PAM-MH may be a useful tool for tailoring and differentiating mental health care for patients with severe, long-term conditions, identifying those who need additional support to become more engaged in their care and helping them to strengthen their activation skills. This is in line with studies [1, 51] suggesting that measuring patient activation is valuable for improving patient health-outcomes and supporting behavioural change. Furthermore, greater understanding of patient activation will allow clinicians to take a more patient-oriented approach to treatment [52]. However, studies are needed to explore if the PAM-MH is valuable for identifying the needs of patients and the most effective mental-health interventions to meet those specific needs. Our study indicates that the PAM-MH may be a useful measure of patient activation in populations with severe mental disorders, as well as in societies other than the United States, such as Norway.

### Limitations

Regarding the face-validity study, we carefully and anonymously collected data in paper format during the first phase of the study to ensure the inclusion of– and respect for– all voices and to provide contextually appropriate questions. However, the small sample size and focus on a single region in Norway constrain this participatory research and limit the generalisability of the findings. This study did not perform confirmatory factor analysis or exploratory factor analysis to assess the dimensionality of the Norwegian version of the PAM-MH scale. The original validation of the PAM-MH [4] included a psychometric analysis using Rasch analysis, which revealed a slightly larger difficulty range compared to the PAM. Moreover, the infit and outfit statistics for the 13-item PAM-MH were all within the acceptable range of 0.5–1.5, and the scores were very similar to those of the original PAM. Given this previous validation study, we recognise that factor analysis would further strengthen the psychometric validation of the PAM-MH. However, we were limited by the sample size requirements specified in the COSMIN guidelines for systematic reviews of patient-reported outcome measures [53]. These guidelines recommend having at least seven participants per item to ensure the stability and reliability of the factor structure. AS the PAM-MH has 13 items, we would need at least 91 participants. However, despite this limitation, our study provides valuable initial validation of the PAM-MH scale.

The process used to find patients was intended to include a variety of people from different backgrounds. However, most of the patients lived at home, needed community care, and had a long-term illness. This could have affected how representative our cohort was and how widely the results could be applied. Another limitation is the short test–retest interval, as the recommended interval is 10–14 days [54]. While the study was intended to validate the PAM-MH in a clinical setting, the results are not intended to be generalised to all age groups but rather to provide insights into how the scale performs in this specific context. Furthermore, a limitation of our findings is in relation to the age distribution of the participant cohort, which was heavily skewed towards middle-aged individuals. The user representatives ranged from 40 to 54 years old. In addition, the mean age for patients with schizophrenia spectrum disorders was 35 years. However, it is worth noting that this is in line with previous studies conducted in Norway, with the mean age being 35 years or older [23, 55, 56]. Patient activation might be influenced by age. Younger patients might be in the earlier stages of their condition, while older patients might have had more time to develop coping strategies and engage with healthcare services, affecting their activation levels. Future studies could build upon this preliminary work and include a more diverse age distribution to ensure the PAM-MH’s applicability across the lifespan. This could involve targeted recruitment strategies to engage younger and older populations. Notably, to our knowledge, this is the first validation study conducted outside of the United States. Although we did not have a large number of previous studies at our disposal with which to compare the results, our findings are strengthened by the fact that they are in accordance with previous existing studies on both the PAM-MH and the PAM-13.

In validation studies, the issue of external validity arises, especially in the present study in which the participants were mostly middle-aged individuals with schizophrenia-spectrum disorders. This context raises questions about whether our findings can be applied to other age groups or individuals with different mental health conditions. Different age groups could lead to varying interpretations of what “activation” means, and similarly, outpatients with different mental health disorders might have different conceptualisations of activation.

## Conclusion

This study took a participatory approach to validating a scale, underscoring the value of collaborative participation in validation studies to ensure both construct validity and face validity. The PAM-MH displayed good face, reliability, and responsiveness-to-change, indicating that it is suitable for measuring patient activation in mental-health settings. Therefore, we suggest it is a useful research tool for measuring patient activation and identifying the effectiveness of interventions. Nonetheless, more studies are needed to compare improvements in the PAM-MH with other measures of progress. Further research is also required for other populations.

## Electronic supplementary material

Below is the link to the electronic supplementary material.

Supplementary Material 1

## Data Availability

The data from this study can be made available to the corresponding author upon reasonable request. The data are not publicly available due to ethical reasons.
