# Supplementary material for: The mental-health patient-activation measure: assessing validity, reliability, and responsiveness in outpatient settings
Source: BMC Psychiatry. 2025 May 22;25:520. doi: 10.1186/s12888-025-06939-5 (PMC12096637; doi:10.1186/s12888-025-06939-5)
Supplement: Supplementary file 1 — Supplementary Material 1 [file 12888_2025_6939_MOESM1_ESM.docx]

GRIPP2 short form

| Section and topic | Item | Reported on page No |
| --- | --- | --- |
| 1: Aim | Report the aim of PPI in the study | 6 |
| 2: Methods | Provide a clear description of the methods used for PPI in the study | 6-7 |
| 3: Study results | Outcomes—Report the results of PPI in the study, including both positive and negative outcomes | 9 |
| 4: Discussion and conclusions | Outcomes—Comment on the extent to which PPI influenced the study overall. Describe positive and negative effects | 13 |
| 5: Reflections/critical perspective | Comment critically on the study, reflecting on the things that went well and those that did not, so others can learn from this experience | 14 |

PPI=patient and public involvement
